# Insights into the genome of the ‘Loco’ *Concholepas concholepas* (Gastropoda: Muricidae) from low-coverage short-read sequencing: genome size, ploidy, transposable elements, nuclear RNA gene operon, mitochondrial genome, and phylogenetic placement in the family Muricidae

**DOI:** 10.1186/s12864-023-09953-7

**Published:** 2024-01-19

**Authors:** J. Antonio Baeza, M. Teresa González, Julia D. Sigwart, Carola Greve, Stacy Pirro

**Affiliations:** 1 Department of Biological Sciences, Clemson University, Clemson, SC USA; 2 Departamento de Biología Marina, Universidad Catolica del Norte, Coquimbo, Chile; 3 Smithsonian Marine Station at Fort Pierce, Smithsonian Institution, Fort Pierce, FL USA; 4 Facultad de Ciencias del Mar y Recursos Biológicos, Instituto de Ciencias Naturales Alexander Von Humboldt, Universidad de Antofagasta, Angamos 601 Antofagasta, Chile; 5 Marine Zoology Department, Senckenberg Research Institute and Museum, Frankfurt, Germany; 6 LOEWE -Centre for Translational Biodiversity Genomics (LOEWE-TBG), Senckenberganlage 25, Frankfurt Am Main, Germany; 7 Iridian Genomes, Silver Spring, MD USA; 8 Institute of Ecology, Evolution & Diversity, Goethe University, Frankfurt, Germany; 9 Senckenberg Forschungsinstitut und Naturmuseum, Frankfurt am Main, Germany

**Keywords:** Genome survey sequencing, Low-coverage genome sequencing, Genome skimming, Snail, Transposable elements, Mitochondrial genome, Neogastropoda, Phylogeny

## Abstract

**Background:**

The Peruvian ‘chanque’ or Chilean ‘loco’ *Concholepas concholepas* is an economically, ecologically, and culturally important muricid gastropod heavily exploited by artisanal fisheries in the temperate southeastern Pacific Ocean. In this study, we have profited from a set of bioinformatics tools to recover important biological information of *C. concholepas* from low-coverage short-read NGS datasets. Specifically, we calculated the size of the nuclear genome, ploidy, and estimated transposable elements content using an in silico k-mer approach, we discovered, annotated, and quantified those transposable elements, we assembled and annotated the 45S rDNA RNA operon and mitochondrial genome, and we confirmed the phylogenetic position of *C. concholepas* within the muricid subfamily Rapaninae based on translated protein coding genes.

**Results:**

Using a k-mer approach, the haploid genome size estimated for the predicted diploid genome of *C. concholepas* varied between 1.83 Gbp (with kmer = 24) and 2.32 Gbp (with kmer = 36). Between half and two thirds of the nuclear genome of *C. concholepas* was composed of transposable elements. The most common transposable elements were classified as Long Interspersed Nuclear Elements and Short Interspersed Nuclear Elements, which were more abundant than DNA transposons, simple repeats, and Long Terminal Repeats. Less abundant repeat elements included Helitron mobile elements, 45S rRNA DNA, and Satellite DNA, among a few others.The 45S rRNA DNA operon of *C. concholepas* that encodes for the ssrRNA, 5.8S rRNA, and lsrRNA genes was assembled into a single contig 8,090 bp long. The assembled mitochondrial genome of *C. concholepas* is 15,449 bp long and encodes 13 protein coding genes, two ribosomal genes, and 22 transfer RNAs.

**Conclusion:**

The information gained by this study will inform the assembly of a high quality nuclear genome for *C. concholepas* and will support bioprospecting and biomonitoring using environmental DNA to advance development of conservation and management plans in this overexploited marine snail.

**Supplementary Information:**

The online version contains supplementary material available at 10.1186/s12864-023-09953-7.

## Background

Among gastropod molluscs, known because of their species-richness and eco-morphological disparity ([1] Aktipis et al. 2018), the Peruvian ‘chanque’ or Chilean ‘loco’ *Concholepas concholepas* (Bruguière, 1789) represents an interesting case of shell form evolution—it exhibits a flattened rather simple limpet-like shell in a family characterized by spectacularly ornamented spiral shells ([2] Vermeij 2017). *Concholepas concholepas* is also an economically, ecologically, and culturally important muricid (Muricidae) heavily exploited by artisanal and commercial fisheries along most of its range of distribution in the temperate southeastern Pacific Ocean ([3] Manriquez and Castilla 2018).

**Fig. 1:**
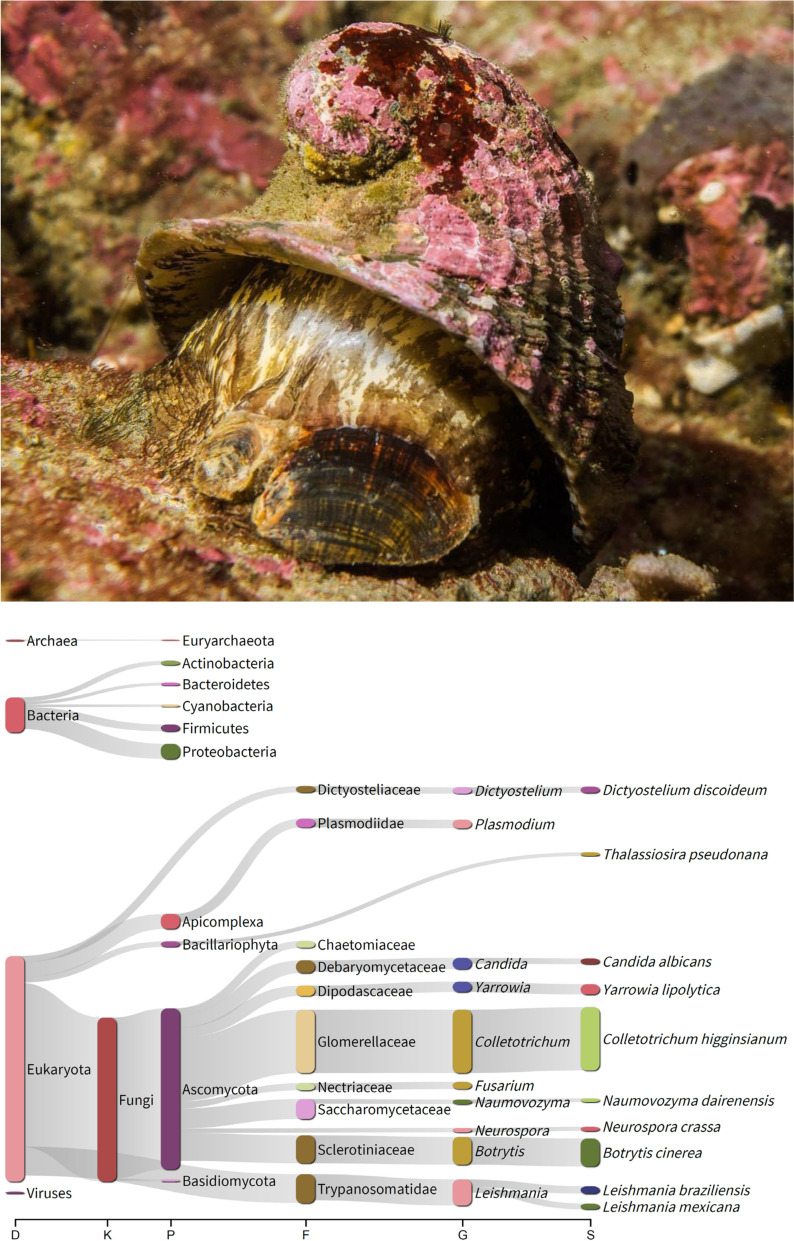
The Chilean ‘loco’ *Concholepas concholepas* (top) and sankey diagram generated from the Kraken2 results obtained for *Concholepas concholepas* (bottom). Photograph credit: Cristian Sepulveda (published with permission)

The species inhabits cold and temperate waters in the southwestern coast of South America, from Lobos de Afuera in Peru to Cape Horn in Chile (Fig. 1). *Concholepas concholepas* is also present in the Juan Fernandez Archipelago, off the central coast of Chile. This carnivorous snail lives in intertidal and shallow subtidal rocky habitats among holdfasts in kelp forests or in encrusting communities composed of mussels, tunicates, and/or barnacles ([4] Stotz et al. 2003, [3] Manríquez and Castilla 2018). *Concholepas concholepas* is considered a keystone species in rocky shores; it controls (via consumption) the abundance of the competitive dominant mussel *Perumytilus purpuratus* and thus liberates primary space for barnacles and algae to grow. Overall, the diversity of benthic primary-substratum users increases in the presence of *C. concholepas* ([5] Castilla 1999). Adult specimens of the edible *C. concholepa*s can reach a maximum shell length of 150–160 mm ([6] Wolff 2008) and have been heavily targeted together with juveniles by subsistence and artisanal fisheries for at least 60 years in the southeastern Pacific coast ([3] Manriquez and Castilla 2018). Currently, *C. concholepa*s is one of the main invertebrates targeted by small-scale fisheries with territorial use rights in Chile but it has been harvested by coastal human societies for more than 8–10 thousand years in Peru and Chile ([7] Jerardino et al. 1992, [8] Reitz et al. 2017, [9] Santoro et al. 2017).

Given its ecological role and commercial importance, the life history and population dynamics of *C. concholepas* are relatively well studied ([10] Molinet et al., 2005, [3] Manriquez & Castilla 2018, and references therein) and the species has been used as a model system during the last decades in research focusing on population and community ecology, ecophysiology, behavioral ecology, genetics and molecular biology, and biogeography, among others (e.g. [3] Manriquez and Castilla 2018 and references therein). Unexpectedly, despite the ecological relevance, cultural significance, and commercial value of *C. concholepas*, only a few genomic resources have been developed for this species (i.e., [11–13] Cárdenas et al. 2007, 2011, 2016, [14] Núñez-Acuña et al. 2013, [15] Gallardo-Escárate et al. 2013, [16] Détrée et al. 2017). Advancing genomic resources in this iconic snail is of utmost importance to continue improving the understanding of its ecology and key role in its community while also supporting conservation and fisheries management plans.

The present study forms part of a comprehensive project to develop genomic resources for *C. chocholepas* and other marine organisms that are intensively targeted by commercial and artisanal fisheries in the temperate Southeastern Pacific Ocean. Here, we have used low-coverage short read next generation sequencing and profited from a set of bioinformatic pipelines designed to retrieve biological information from low-coverage datasets to i. estimate the genome size and ploidy of *C. chocholepas* using an in silico k-mer strategy, ii. calculate the content of transposable elements in the nuclear genome of *C. chocholepas*, iii. annotate and characterized those transposable elements, iv. assemble the 45S rRNA nuclear DNA operon that encodes the large and small nuclear rRNA genes (18S or ssrDNA, 28S or lsrDNA), the 5.8S rDNA gene, two internal transcribed spacers (ITS1 and ITS2), and two external transcribed spacers (5′ ETS and 3′ ETS), v. assemble, annotate, and describe in detail the mitochondrial genome (mitogenome) of *C. concholepas*, and explore the position of *C. concholepas* among muricid gastropods based on the phylogenetic signal provided by translated protein coding genes. These new genomic resources will guide a chromosome-level genome assembly of *C. chocholepas* and will eventually support fisheries management and conservation strategies in this heavily fished and keystone edible muricid from the temperate southeastern Pacific Ocean.

## Results and discussion

### Genome size and ploidy estimation in *Concholepas concholepas*

Using an in silico k-mer approach, the haploid genome size estimated for *C. concholepas* varied between a minimum of 1,825,342,588 bp (1.83 Gbp, with kmer = 24) and a maximum of 2,327,023,338 bp (2.32 Gbp, with kmer = 36). No clear trend of concomitant increases in genome size with k-mer word size was observed in our analysis. Genome size (GS) estimated using either flow cytometry, Feulgen densitometry, bulk fluorometric assay, or biochemistry analysis is known for only 13 muricid gastropods (Animal Genome Size Database [http://www.genomesize.com/] – [17] Gregory 2021 [consulted on 9 8 2023]) and ranges between a minimum of 2.04 Gb in the Southern oyster drill *Thais haemastoma* and 5.75 Gb in the Antarctic whelk *Neobuccinum eatoni* ([17] Gregory 2021). In turn, among the few gastropods with chromosome-level assembled genomes, GS varies between 404,610,835 bp in the Scaly-foot *Chrysomallon squamiferum* ([18] Sun et al. 2022) and 3,592,060,885 bp in the Mediterranean cone *Conus ventricosus* ([19] Pardos-Blas et al. 2021) when calculated using a k-mer strategy or from the assembly size. GS has been estimated only in two species belonging to the family Muricidae; the veined rapa whelk *Rapana venosa* (2.3 Gbp estimated from assembly length—[20] Song et al. [2023]) and the Florida rocksnail *Stramonita haemastoma* (2.24 Gbp, estimated from the assembly length [GCA_030674155.1]—[21] Farhat et al. 2023). Overall, our estimates of GS for *C. concholepas* using an in silico k-mer approach are within the range observed for gastropods and very similar to that reported for muricid snails.

**Fig. 2:**
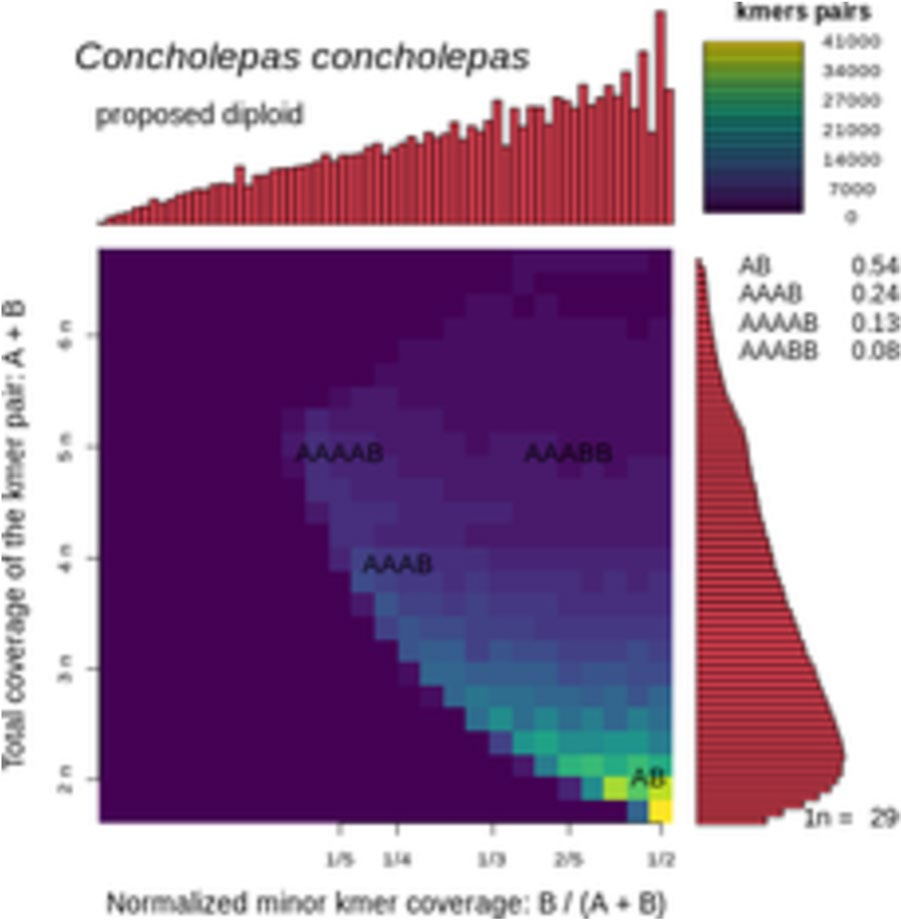
Relationship between coverage of heterozygous k-mer pairs and normalized minor k-mer coverage in *Concholepas concholepas*

Using a second in silico k-mer analysis on the relative abundance of heterozygous k-mer pairs with the program Smudgeplot, the nuclear genome of *C. concholepas* was determined as diploid (Fig. 2). Diploidy is often assumed in muricids and other gastropods, although studies on ploidy are rare in this clade ([22] Lopez et al. 2019). In other gastropods families, species with different ploidy are found within the same family or genus (i.e., in the freshwater snail *Bulinus truncatus* / *tropicus* species complex—[23] Yusuf et al., 2017). Chromosome evolution studies in the family Muricidae are lacking. We argue that a combination of low-coverage sequencing and k-mer spectra analyses can advance our understanding of ploidy evolution and environmental correlates in marine snails and other marine invertebrates.

### Transposable elements in *Concholepas concholepas*

The pipeline RESPECT estimated that the repetitive genome content of *C. concholepas* ranged from a minimum of 49% (with kmer = 51) to a maximum of 66% (with kmer = 21). In our analysis, a trend of decreasing repetitive genome content was observed with increases in k-mer word size. In general, between half and two thirds of the nuclear genome of *C. concholepas* is composed of transposable elements. Repetitive genome content varies considerably among gastropods and ranges from 11.40% in the freshwater snail *Pomacea caniculata* ([24] Liu et al. 2018) to 49% in *Conus consors* ([25] Andreson et al. 2019). In molluscs, repetitive genome content can be as high as 62% (i.e., in the marine mussel *Modiolus philippinarum*—[26] Sun et al., 2017). Repetitive content is available only for one of the two muricids with assembled genomes; in *Rapana venosa* repetitive content is 57.72%% ([20] Song et al. 2023). Overall, repetitive content in the genome of *C. concholepas* is within the range observed for gastropods and is similar to that reported for the cofamilial *Rapana venosa*. The size of and the high proportion of transposable elements in the nuclear genome of *C. concholepas* suggests that chromosome conformation capture techniques (i.e., Hi-C) in addition to short and long-reads (i.e., Oxford Nanopore Technology and/or Pacific Biosciences) will be necessary to assemble a chromosome-level genome in this gastropod.

**Fig. 3:**
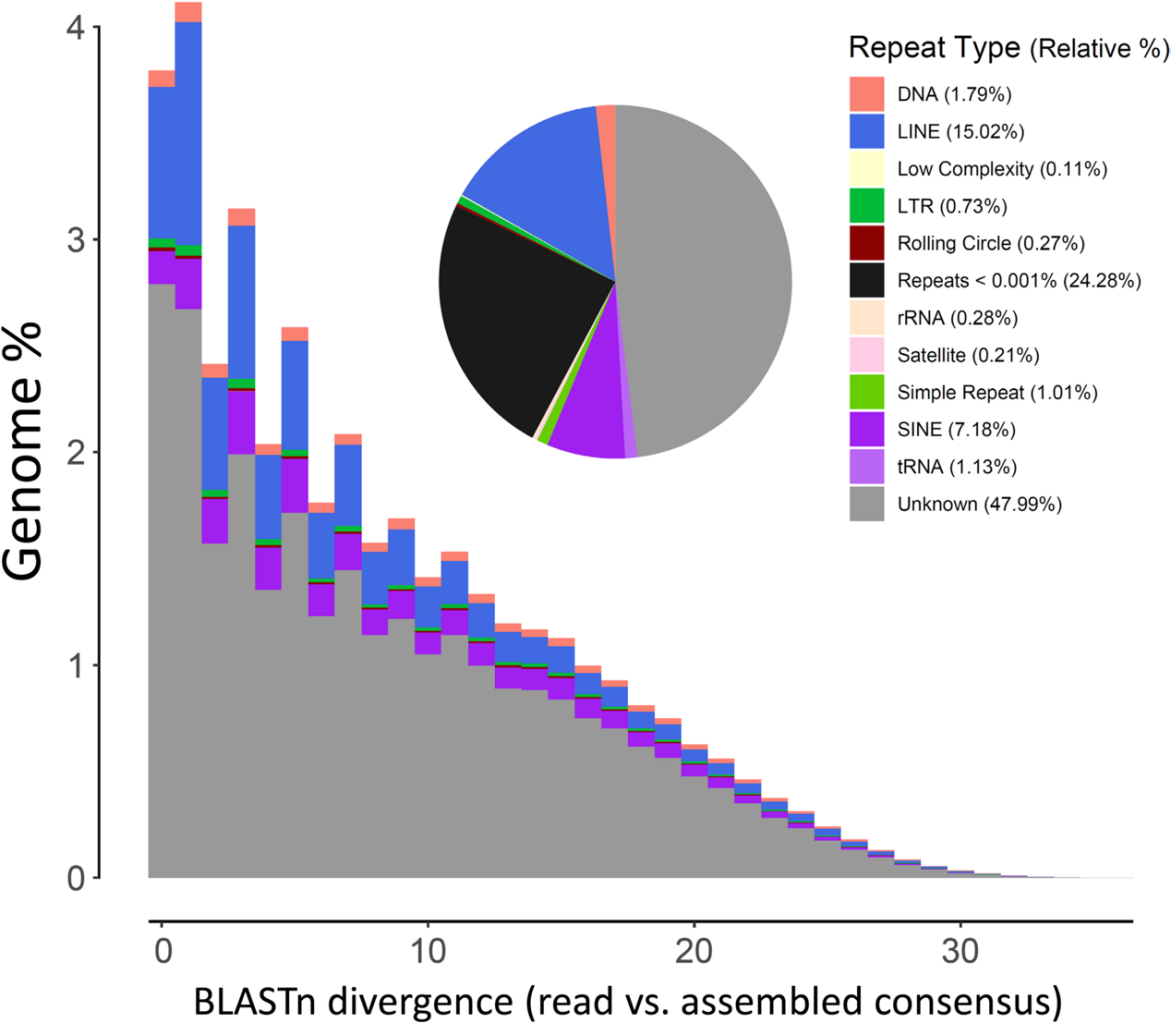
Transposable elements genome composition and landscape in the genome of *Concholepas concholepas*

The program dnaPipeTE estimated that 34.19% of the genome in *C. concholepas* represented repetitive elements, a value lower than that reported by RESPECT. Also, DnaPipeTE reported a relatively high portion of repetitive elements (i.e., 47.99%) as ‘unknown’; these repetitive elements were not annotated (not assigned to any known family) using the Protostomia database of transposable elements from the Dfam consortium. Taking into account only those repetitive elements that were annotated by DnaPipeTE, the most common repetitive elements were classified as Long Interspersed Nuclear Elements (LINEs, 15.12%) and Short Interspersed Nuclear Elements (SINEs, 7.18%), which were more abundant than DNA transposons (DNA, 1.79%), tRNA (1.13%), simple repeats (1.01%) and Long Terminal Repeats (LTR, 0.73%). Less abundant repeat elements included rRNA DNA (0.28%) and Satellite DNA (0.21%), among a few others (Fig. 3). In other gastropods with assembled genomes in which the ‘repeatome’ has been characterized, the proportion of unclassified repetitive elements is usually low, in disagreement with our observations. For example, only 0.16% of the genome content corresponds to unclassified repetitive elements in the cofamilial *Rapana venosa* ([20] Song et al. 2018). Interestingly, in the latter species, LINEs were the most common (39.636% of the assembled genome) but SINEs were the rarest (6.09 Mb, 0.27%) among repetitive elements ([20] Song et al. 2023). No ‘repeatome’ analysis is available for the second muricid with an assembled genome, *Stramonita haemastoma* ([21] Farhat et al. 2023). Expansion of repetitive elements has been suggested to account for genome size increases in both vertebrates and invertebrates ([27] Helmkampf et al. 2019). Whether or not expansion of mobile genetic elements explain genome size variance in gastropods and other molluscs remains to be addressed.

DnaPipeTE also estimated the repetitive elements ‘landscape’ in *C. concholepas* that exhibited a leptokurtic distribution (Fig. 3). However, no obvious ‘peaks’, either in the recent or distant past, were observed in the repetitive elements landscape that could be interpreted as ancient bursts. Still, the analysis suggests that transposable elements have a high turnover in the nuclear genome of *C. concholepas*. By contrast, in the deep-sea limpet *Bathyacmaea lactea* repetitive elements have undergone long-lasting activity in the deep-past (i.e., until the last 10 Mya) that included two concentrated TE expansions ([28] Liu et al. 2020). To the best of our knowledge, no studies have examined the transposable elements landscape in gastropods other than in *C*. *concholepas* (this study) and *Bathyacmaea lactea* ([26] Liu et al. 2020). Future studies focusing on transposable elements activity will permit the exploration of the conditions driving the dynamics of the ‘repeatome’ in the species-rich order Gastropoda. Furthermore, Casacuberta and González (2013) [29] have argued that repetitive elements can influence the capability of their hosts to respond to environmental challenges. Whether repetitive elements affect the ability of molluscs and other marine invertebrates to pervasive global change challenges remains to be addressed.

### *45S rRNA DNA* assembly *inConcholepas concholepas*

The pipeline TAREAN assembled the 45S rRNA DNA operon of *C. concholepas*. The assembled sequence was 8,090 bp long and comprised a 5′ ETS (length = 980 bp [partially assembled]), ssrDNA (1,828 bp [fully assembled, GenBank accession number OR501214]), ITS1 (561 bp), 5.8S rDNA (154 bp [fully assembled, OR509795]), ITS2 (394 bp), lsrDNA (3,894 bp [fully assembled, OR501215]), and 3′ ETS (278 bp [partial sequence]). The assembled operon matched other gastropod partial 18S and 28S ribosomal sequences available in GenBank with E-values <  < 1e^−6^.

Importantly, phylogenetic relationships within and among the different mollusc lineages, including gastropods, have been explored using fragments of the 18S and 28S ribosomal genes for more than 20 years now ([30] Colgan et al. 2007, [31] Zou et al. 2011). We have shown here that low-coverage sequencing data can be used to assemble the complete 45S rRNA DNA operon of *C. concholepas*. The recovery of additional 45S rRNA DNA sequences in other muricids using bioinformatic tools tailored for low-coverage sequencing datasets can be used to understand the organization and evolutionary dynamics of this repetitive element in molluscs.

### The mitochondrial genome of *Concholepas concholepas*

**Fig. 4:**
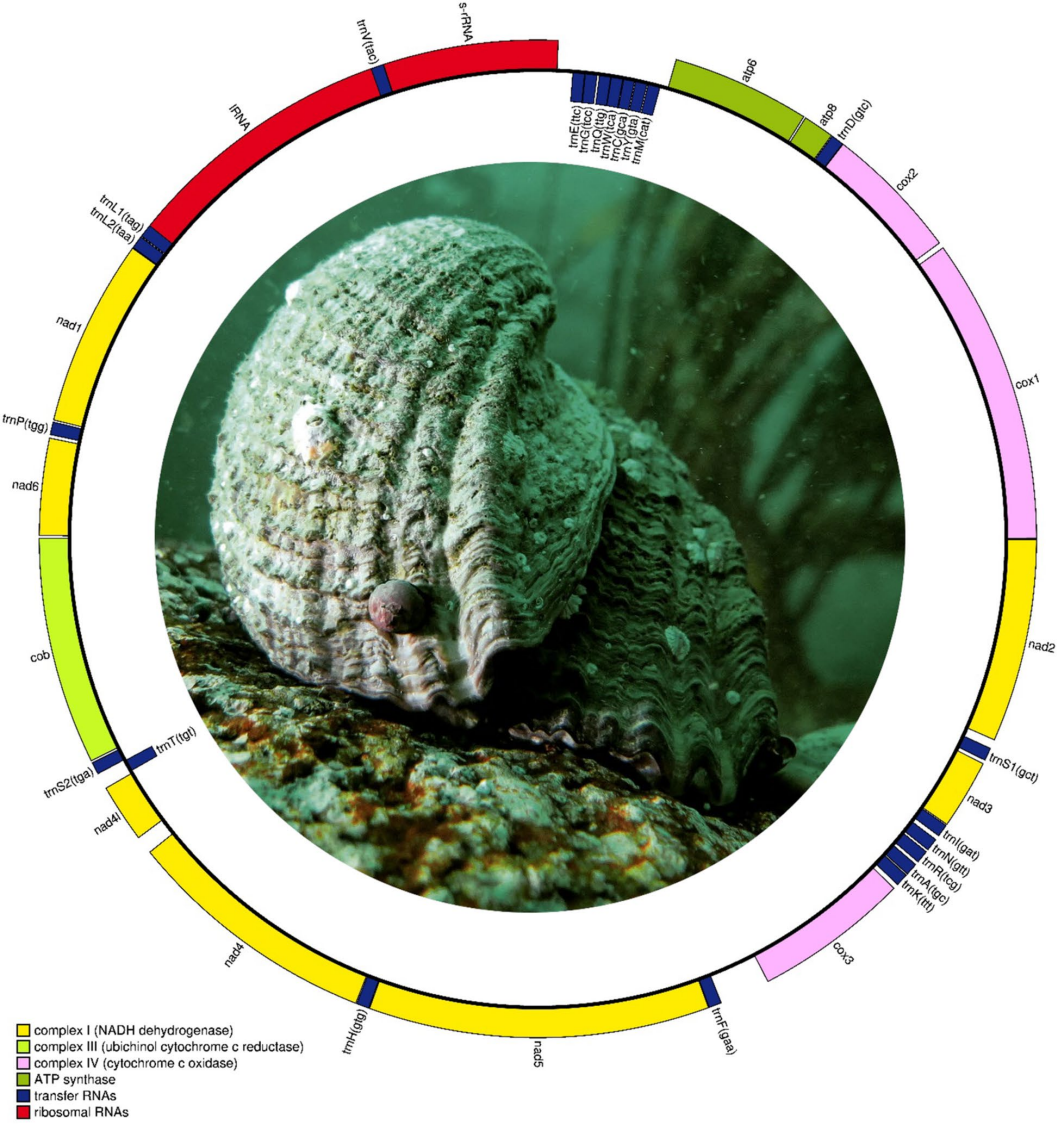
Circular map of the mitochondrial genome of *Concholepas concholepas*. Photograph credit: Gustavo Duarte (published with permission)

The pipeline GetOrganelle assembled the mitochondrial genome of *C. concholepas* (OR506260) with a k-mer- and base-coverage equal to 125 × and 521x, respectively. The mitochondrial genome of *C. concholepas* is 15,449 bp long and encoded 13 protein-coding genes (PCGs), 22 transfer RNA (tRNA) genes, and two ribosomal RNA genes (12S ribosomal RNA [rrnS] and 16S ribosomal RNA [rrnL]) (Table 1 Fig. 4). The mitochondrial genome of *C. concholepas* also contains a relatively short non-coding putative Control Region (CR) 249 bp long. Mitochondrial gene order in *C. concholepas* was identical to that previously reported for other species of gastropods belonging to the family Muricidae ([32] Cunha et al. 2009, [33] Yu et al. 2023) with the exception of *Coralliophila richardi* which exhibits a derived mitochondrial synteny from the neogastropod ground pattern due to the deletion of one tRNA gene as well as the translocation of various other tRNA genes and *apt8* ([34] Harasewych et al. 2022).

**Table 1:** Mitochondrial genome of *Concholepas concholepas*. Arrangement and annotation

Name	Start	Stop	Strand	Length	Start/Stop Codons	Continuity
*cox1*	1	1533	+	1533	ATG/TAA	25
*cox2*	1559	2245	+	687	ATG/TAA	-2
trnD(gtc)	2244	2311	+	68		1
*atp8*	2313	2471	+	159	ATG/TAA	6
*atp6*	2478	3173	+	696	ATG/TAA	37
trnM(cat)	3211	3278	-	68		2
trnY(gta)	3281	3346	-	66		1
trnC(gca)	3348	3410	-	63		0
trnW(tca)	3411	3476	-	66		-2
trnQ(ttg)	3475	3539	-	65		8
trnG(tcc)	3548	3614	-	67		-1
trnE(ttc)	3614	3680	-	67		79
rrnS	3760	4638	+	879		-3
trnV(tac)	4636	4703	+	68		-17
rrnL	4687	6060	+	1374		-7
trnL1(tag)	6054	6123	+	70		1
trnL2(taa)	6125	6193	+	69		0
*nad1*	6194	7135	+	942	ATG/TAA	6
trnP(tgg)	7142	7209	+	68		13
*nad6*	7223	7711	+	489	ATT/TAA	8
*cob*	7720	8859	+	1140	ATG/TAA	7
trnS2(tga)	8867	8931	+	65		2
trnT(tgt)	8934	8999	-	66		9
*nad4l*	9009	9305	+	297	ATG/TAG	86
*nad4*	9392	10,669	+	1278	ATG/TAA	1
trnH(gtg)	10,671	10,737	+	67		0
*nad5*	10,738	12,447	+	1710	ATG/TAG	-1
trnF(gaa)	12,447	12,514	+	68		249
*cox3*	12,764	13,543	+	780	ATG/TAA	21
trnK(ttt)	13,565	13,632	+	68		3
trnA(tgc)	13,636	13,702	+	67		13
trnR(tcg)	13,716	13,784	+	69		8
trnN(gtt)	13,793	13,860	+	68		19
trnI(gat)	13,880	13,946	+	67		2
*nad3*	13,949	14,302	+	354	ATG/TAA	15
trnS1(gct)	14,318	14,385	+	68		42
*nad2*	14,428	15,447	+	1020	ATG/TAG	2
CR	15,448					0

The nucleotide composition of the positive DNA strand in the mitochondrial genome of *C. concholepas* was T = 42.2%, A = 36.6%, G = 18.3%, and C = 17.4%. The A + T rich content (= 79.8%) in the studied mitochondrial genome is similar to that reported for other muricid gastropods ([35] Zhong et al. 2020, [34] Harasewych et al. 2022, [33] Yu et al. 2023). All mitochondrial PCGs started with the codon GTA with the exception of *nad6* that used the start codon ATA. Also, PCGs preferentially ended with the stop codon TAA and only 3 genes (*nad2, nad4l* and *nad5*) terminated with the stop codon TAG. The use of start and stop codons by the PCGs of *C. concholepas* is similar to that reported in other cofamilial gastropods ([35] Zhong et al. 2020, [33] Yu et al. 2023).

**Fig. 5:**
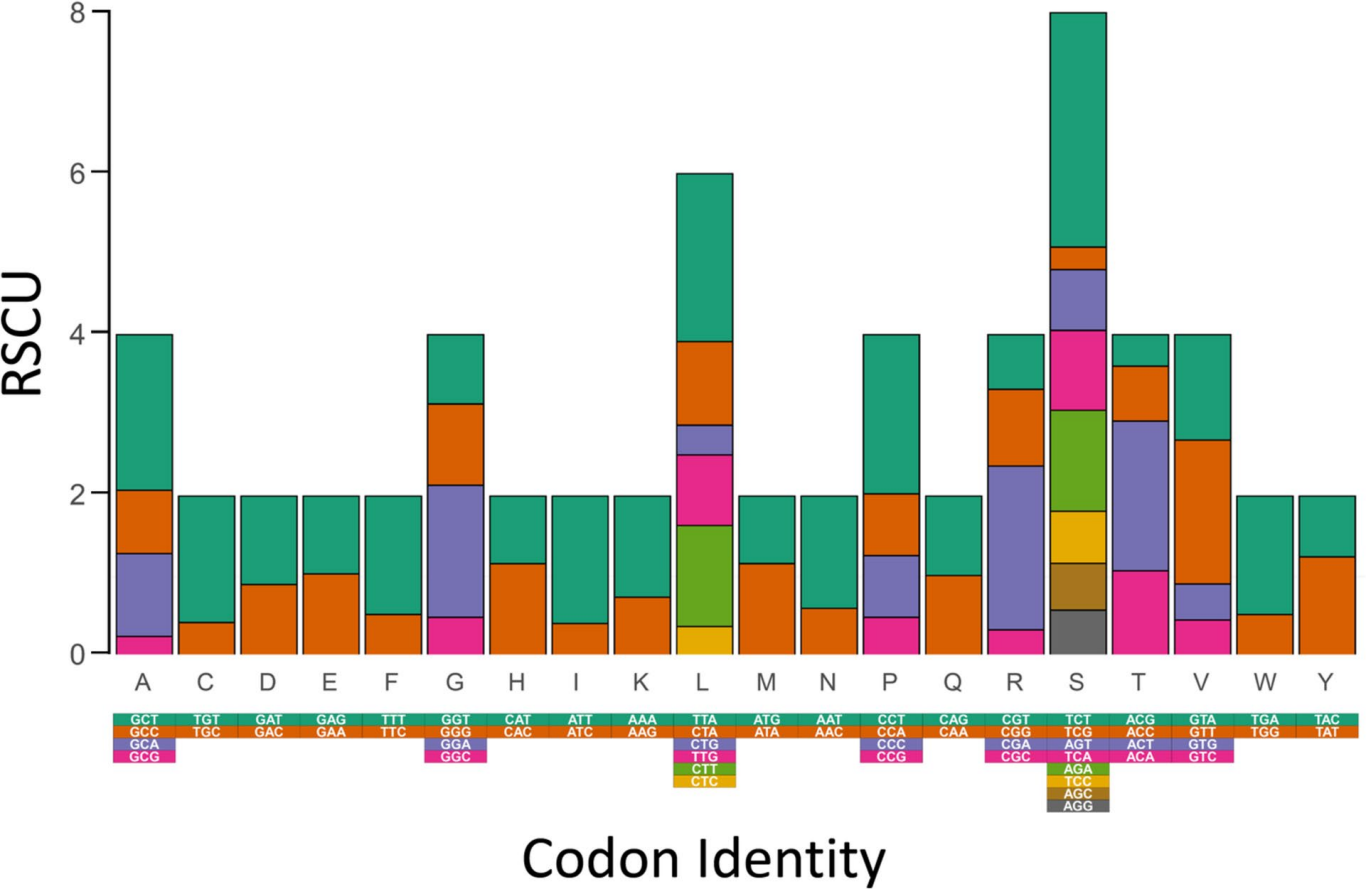
Relative synonymous usage in the 13 protein coding genes encoded in the mitochondrial genome of *Concholepas concholepas*

In the mitochondrial PCGs of *C. concholepas*, codons were not used proportionally. The most frequently used codons (> 100 times) were ATT (Ile, used 220 times), TTT (Phe, *n* = 236), TTA (Leu, *n* = 203), TCT (Ser, *n* = 135), CTT (Leu, *n* = 122), GCT (Ala, *n* = 120), GTT (Val, *n* = 120), ATA (Met, *n* = 108), GCA (Gly, *n* = 103), and CTA (Leu, *n* = 101). In turn, excluding stop codons, the least frequently used codons (< 20 times) were CCG (Pro, *n* = 18), ACG (Thr, *n* = 16), GCG (Ala, *n* = 16), CGG (Arg, *n* = 14), TCG (Ser, *n* = 13), TGC (Cys, *n* = 9), and CGC (Arg, *n* = 5) (Supplementary Materials Table S1). Each amino acid in the mitochondrial PCGs of *C. concholepas* was encoded by a minimum of two or a maximum of 8 codons with the former being more typical (12 out of 20 amino acids) (Fig. 5). RSCU values also indicated that all the (synonymous) codons for the same amino acid were not used equally in the mitochondrial PCGs of *C. concholepas*. Specifically, codons ending in A or T were overrepresented compared to codons ending in C or G (Fig. 5). Studies on the codon usage of mitochondrial PCGs have not been conducted before in other muricid gastropods. However, codon usage biases in mitochondrial PCGs have been invariably reported in other marine invertebrates, including molluscs (e,g., in bivalves – [36] Sun and Gao 2017) and gastropods (e.g., in the family Strombidae – [37] Li et al. 2022). The AT-rich nucleotide usage of the studied mitochondrial genome is likely a reflection of the codon usage bias reported herein for the mitochondrial PCGs of *C. concholepas*. The conditions explaining the non-random use of codons in mitochondrial PCGs are not well understood and several factors have been proposed to drive genome-wide or mitogenomic codon usage biases i.e., mutational bias, selection for optimizing the translation process by tRNA abundance, and harsh environmental conditions, among others (see [38] Jia and Higgs 2008 and references therein).

The *w* ratios calculated for all the PCGs in the mitochondrial genome of *C. concholepas* were lower than 1 (Table 2), implying that all of these genes experience purifying selection. The highest *w* values were observed in *atp8, cox3,* and all PCGs belonging to the *nad* family except *nad1* indicating that the aforementioned genes (other than *nad1*) are experiencing the weakest selective pressures in the mitochondrial genome of *C. concholepas*. In turn, the lowest *w* values were observed in *cox1, cox2, cob, nad1, and atp6*, indicating that these genes are experiencing the strongest selective pressure compared to the remainder PCGs. Selective pressures analyses have not been conducted before in any representative of the family Muricidae. However, our results agree with those from studies in other gastropod clades showing that all PCGs are under purifying selection (e.g., in the family Neritidae [[39] Feng et al. 2021] and Strombidae [37] [Li et al. 2022], among others). The species richness and ecological disparity characteristic of the Muricidae suggest that this family might be a suitable model system to understand the effect of environmental conditions on the adaptive evolution of mitochondrial protein coding genes.

**Table 2:** Selective pressure analysis of the protein coding genes (PCGs) in the mitochondrial genome of *Concholepas concholepas*. K_A_/K_S_ values were calculated using the γ-MYN model using the mitochondrial genome of *Rapana venosa* as outgroup

PCG	dN	dS	*w*	*p*-value
*atp6*	0.026312	3.74052	0.007034	1.27E-94
*atp8*	0.034173	1.6343	0.02091	8.98E-11
*cob*	0.021608	4.11164	0.005255	4.04E-160
*cox1*	0.005649	4.23484	0.001334	6.17E-255
*cox2*	0.003628	5.80398	0.000625	6.41E-87
*cox3*	0.022729	1.4095	0.016126	4.15E-75
*nad1*	0.010746	3.91741	0.002743	2.87E-122
*nad2*	0.081797	1.67447	0.04885	2.02E-82
*nad3*	0.046626	1.592	0.029288	7.88E-26
*nad4*	0.029794	1.61022	0.018503	7.87E-134
*nad5*	0.056762	2.52031	0.022522	2.73E-162
*nad6*	0.072921	3.43793	0.021211	1.74E-65
*nad4l*	0.023477	0.871114	0.026951	9.17E-20

**Fig. 6:**
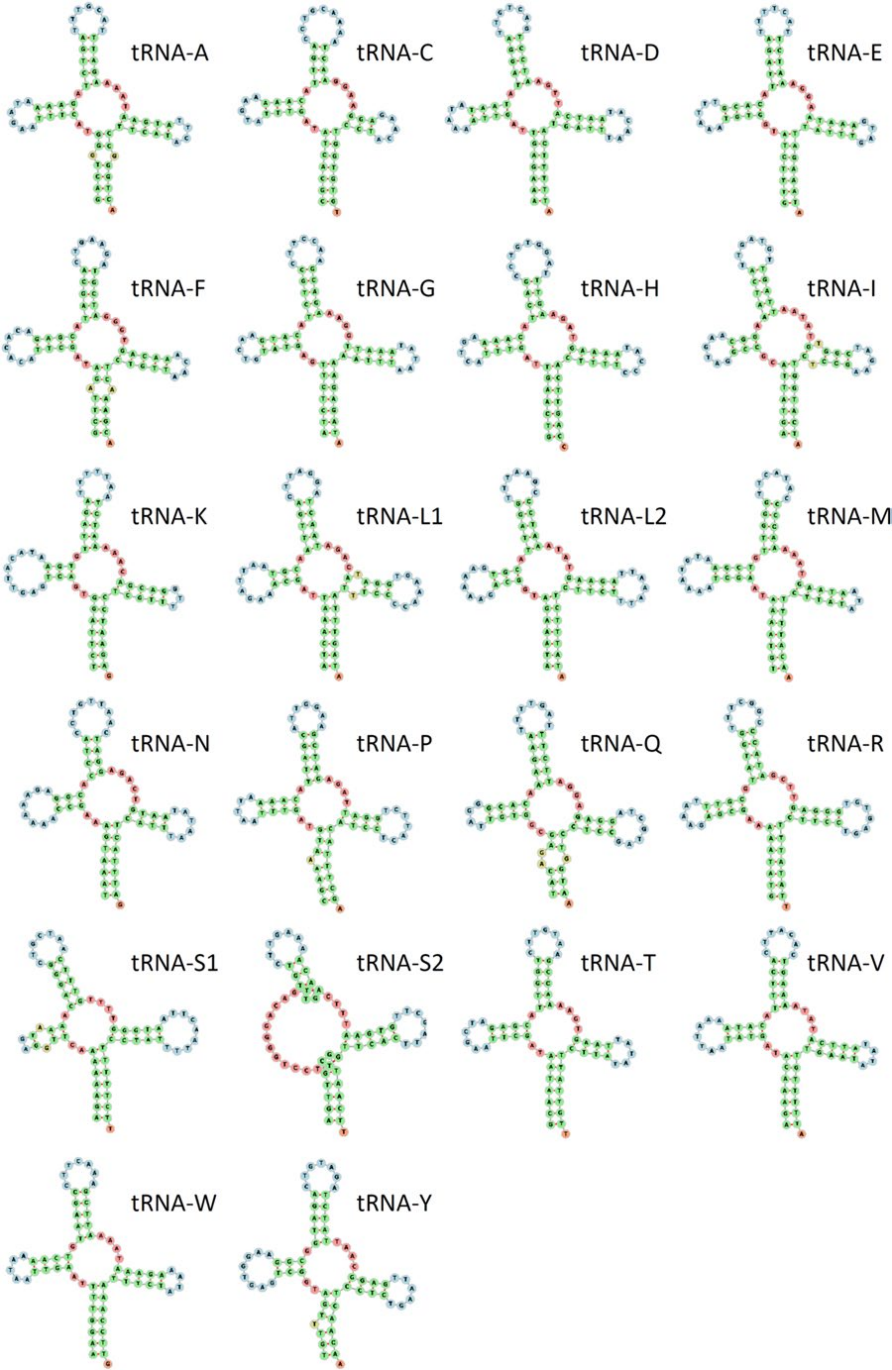
Secondary structure of the tRNA genes encoded in the mitochondrial genome of *Concholepas concholepas*

In the mitochondrial genome of *C. concholepas*, the 22 tRNAs varied in length between 65 bp (trnS2) and 70 bp (trnL1). All tRNAs exhibited a typical 'cloverleaf' secondary structure except trnS1 and trnS2, which lacked the DHU arm and loop, respectively (Fig. 6). To the best of our knowledge, no previous study has examined the secondary structure of mitochondrial tRNA genes in muricid gastropods. Nonetheless, truncated tRNA genes for Serine are commonly reported in eumetazoans ([40] Bernt et al. 2013), and trna-S1 has been shown to be truncated in the few gastropods in which the secondary structure of these genes have been examined ([41] Wang et al. 2022, [42] Xu et al. 2023).

The relatively short (249 bp long) non-coding putative Control Region (CR) in the studied mitochondrial genome is located between trnaF and *cox3* and exhibits a much lower A + T content (59%, with A = 78 [31.3%], T = 69 [27.7%], G = 51 [20.5%], and C = 51 [20.5%]) than that of the entire mitochondrial genome molecule (79.8%). No tandem repeats were found in this region, likely because of its short span. However, two dinucleotide-motif microsatellite repeats (AA and TT, each repeated 3 times) were reported by the web server Microsatellite Repeat Finder. Lastly, the web server RNAFold predicted a single optimal secondary structure with a minimum free energy of -212.00 kcal/mol (free energy of the thermodynamic ensemble = -212.34 kcal/mol) that formed a single ‘hairpin’ bearing a long stem and very short loop (Supplementary Materials Fig. S1). The long stem was the consequence of a perfect 111 bp long palindromic motif (5’- AGC CAG CAC TCA CTC CAA GAG TGC TGG CCA AAG GGC TCC GCC GAG CGA ACC TGA AAT TTT ATA GTT TTA GAG GCA CAG AGC CAA AAT TAT CTA TTT TTT GCT TAA TTT CTA—3’. Importantly, the non-coding putative CR in the mitochondrial genome of muricids and other gastropods is short and can be considered extremely truncated compared to that of other marine invertebrates (see [33] Yu et al. 2023). Detailed analyses of this non-coding region in gastropods mitochondrial genomes is rare ([33] Yu et al. 2023). We argue that additional studies characterizing this region in detail will help us understand mitochondrial genome replication and translation in gastropods.

### Phylomitogenomics of the family Muricidae

**Fig. 7:**
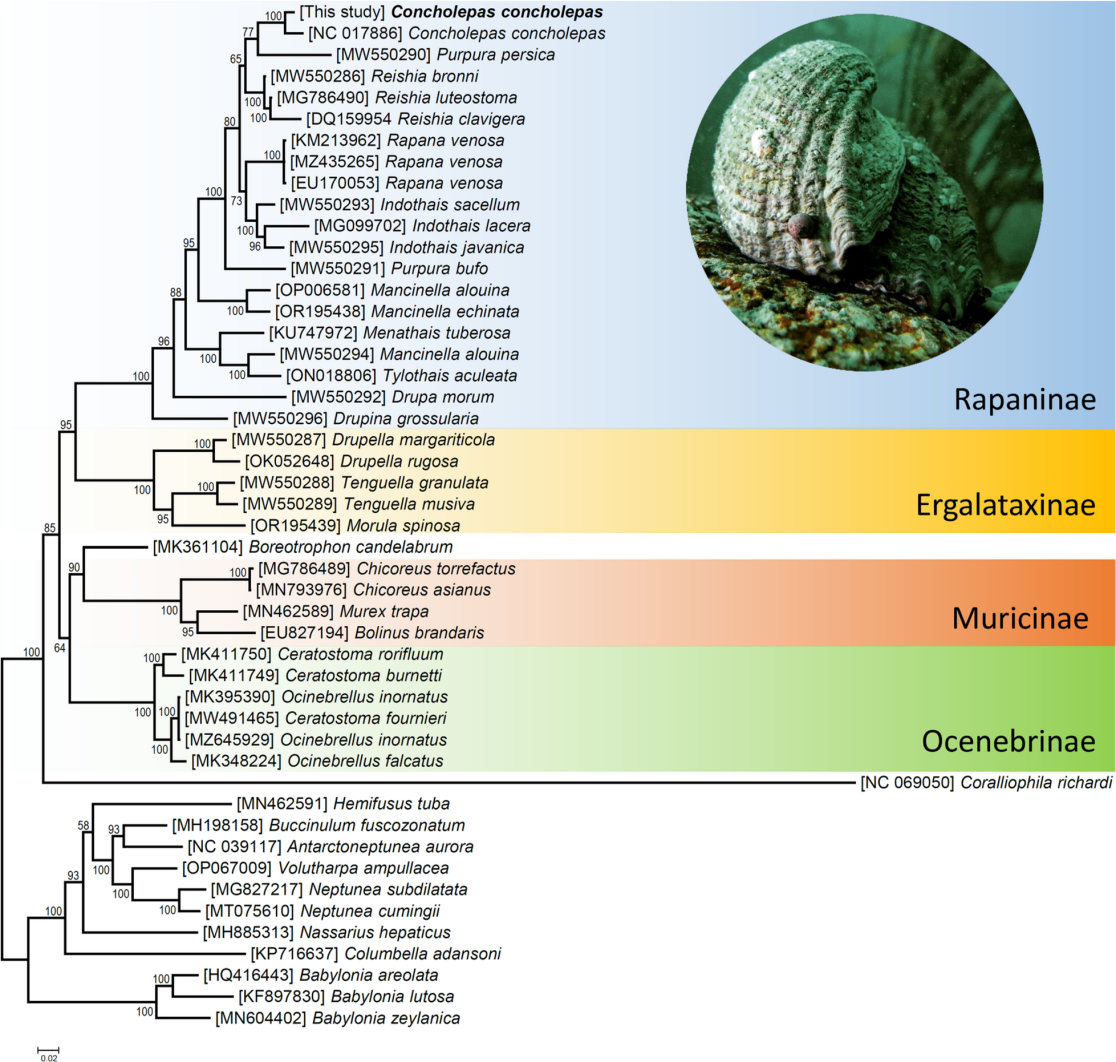
Maximum likelihood phylogenetic hypothesis for the family Muricidae and phylogenetic placement of *Concholepas concholepas*. The tree was retrieved using the phylogenetic signal provided by the translated mitochondrial protein coding genes. The robustness of the ML tree topology was ascertained by 1,000 boot Numbers above branches near nodes represent bootstrap pseudoreplicates of the tree search. Photograph credit: Gustavo Duarte (published with permission)

In the ML phylogenetic analysis (48 terminals, 3,697 characters, 1,066 parsimony-informative sites), *C. concholepas* together with all other representatives of the family Muricidae used in this study clustered together into a single fully supported clade (bootstrap value [bv] = 100) (Fig. 7). Within the monophyletic family Muricidae, fully supported subfamilies included the Ergalataxinae, represented by 3 genera and 6 species in our analysis, Ocenebrinae, represented by 2 genera and 6 species, Muricinae, represented by 3 genera and 4 species, and Rapaninae, represented by 10 genera and 17 species. The family Padoludinae, represented by a single species, *Boreotrophon candelabrum*, in our analysis, was well supported (bv = 90) as a taxon sister to the subfamily Muricinae. In turn, *Coralliophila richardi* (subfam. Coralliophilinae) have an early branching position in the family Muricidae; it was sister to all other studied muricids, in line with that reported by [34] Harasewych et al. (2022). In general, most of the relationships among subfamilies in muricids recovered by our ML analysis agree with those previously reported by [34] Harasewych et al. (2022) and [33] Yu et al. (2023), which used a smaller set of mitochondrial genomes for phylogenetic reconstruction.

In the subfamily Rapaninae, *C. concholepas* had a late branching position and formed a monophyletic clade with a second mitochondrial genome of *C. concholepas* already available in GenBank (JQ446041). We note that this previously available mitochondrial genome of *C. concholepas* is a chimeric molecule assembled using ‘noisy’ (= high error-rate) pyrosequencing DNA reads, transcriptomic data from 50 specimens, and Sanger sequencing ([14] Núñez-Acuña et al. 2013).

There is a long history of strong interest in this large globally distributed family, and the family Muricidae was mainly established based on shell and radular characteristics but updated with molecular phylogenetic results (e.g. [43] Barco et al. 2010, [44] Claremont et al. 2013). These advances improved the understanding of plasticity and convergence in some shell characters, and while the taxonomic affinities of many species remain enigmatic, *Concholepas* is placed confidently within the subfamily Rapaninae, which is confirmed by our analyses herein. Researchers working on Muricidae already produced a mitogenome phylogeny based on 23 muricid species but with a smaller set of mitochondrial genomes for phylogenetic reconstruction compared to this study ([33] Yu et al. 2023). Data are lacking to enable phylogenomic analyses with strong taxon sampling for molluscs which can bias results ([45] Sigwart et al. 2021) and it is important to continue to expand taxon sampling, especially for unusual morphologies like *C. concholepas.*

## Conclusion

In summary, we have produced a set of genomic resources for the Chilean ‘loco’ or Peruvian ‘chanque’ *C. concholepas*, a species of considerable ecological, commercial, and cultural importance in the southeastern Pacific Ocean that is experiencing heavy fishing pressure and major environmental challenges (i.e., due to pollution, ocean acidification, and increased temperature). This is the first study focusing on this muricid mollusc that has profited from a set of bioinformatics tools to recover important biological information from low-coverage short-read NGS datasets. We have calculated the size and ploidy of the nuclear genome and estimated its transposable elements content. Also, we have discovered, annotated, and quantified these repetitive elements. We have assembled and annotated the 45S rDNA RNA operon and mitochondrial genome. Lastly, we have confirmed the phylogenetic position of *C. concholepas* in the muricid subfamily Rapaninae based on translated PCGs. The new information generated by this study will inform the assembly of a high quality nuclear genome for *C. concholepas*, is expected to support bioprospecting and biomonitoring using state-of-the-art genomic techniques (eDNA) in this species, and will contribute to improve the understanding of the genomic mechanisms related to the acclimatization of this remarkable mollusc to pollution and the adaptation to pervasive global change.

## Methods

### Specimen, DNA extraction, library preparation and sequencing

A frozen specimen of *C. concholepas* (caught in Chile) was bought from a local supermarket in Raleigh, North Carolina, USA and transported to Clemson University (CU), Clemson, South Carolina, USA. The specimen was deposited at CU’s Crustacean Collection (accession number CU-CC-2022–15-05). In the laboratory, a small tissue fragment (0.75 cm^3^) was dissected from the foot and preserved in 95% ethanol for shipping to Iridian Genomes, Inc. (Bethesda, MD) where genomic DNA (gDNA) extraction and next generation sequencing (NGS) were conducted. gDNA was extracted from the sample using the DNeasy Blood and Tissue Kit (Qiagen, Germany) following the manufacturer’s protocol. Then, library preparation was constructed using the Illumina TruSeq kit following the manufacturer’s protocol. NGS was performed in a Illumina HiSeq X Ten system (Illumina, San Diego, CA, USA) using a 2 × 150 cycle. A total of 72,006,895 pairs (PE) of reads were produced by Iridian Genomes and were deposited in the short read archive (SRA) repository (Bioproject: PRJNA996197; BioSample: SAMN36527401; SRA accession number: SRR25338493) at NCBI’s GenBank.

### Genome size and ploidy estimation in *Concholepas concholepas*

To estimate genome size in *C. concholepas* using an in silico k-mer strategy, we first removed Illumina adapters and low quality sequences (Phred scores < 20) from the dataset (raw Illumina reads) using the program fastp v.0.20.1 with default options ([46] Chen et al. 2018). Out of 72,006,895 PE raw reads, a total of 68,310,389 high quality remaining PE reads remained, from which contaminants (virus, archaea, bacteria, and human reads) were removed with the pipeline Kraken2 v2.1.2 ([47] Wood et al. 2019) using the pre-built database kraken2-microbial-fatfree (https://lomanlab.github.io/mockcommunity/mc_databases.html) (Fig. 1). A total of 61,894,924 high quality and contaminant-free PE reads were used for calculating genome size of *C. concholepas* with the pipeline KMC 3 v. 3.2.1 ([48] Kokot et al. 2017) using k-mers of 11 different word lengths (= 21, 24, 27, 30, 33, 36, 39, 42, 45, 48, 51) following [49] Baeza et al. (2023). The program RESPECT (REPeat SPECTra Estimation) v.1.0.0 ([50] Sarmashghi et al., 2021) was used to analyze the k-mer frequency distribution and estimate genome size in *C. concholepas*.

To estimate ploidy in *C. concholepas* using an in silico k-mer strategy, the k-mer frequency distribution generated with the pipeline KMC using word size equal to 21 was submitted to the program Smudgeplot v0.2.5 ([51] Ranallo-Benavidez et al. 2020). After visual examination of k-mer coverage in the web server GenomeScope (http://qb.cshl.edu/genomescope/genomescope2.0—[51] Ranallo-Benavidez et al. 2020), we selected high coverage k-mers between 20 × and 120 × for the analysis of heterozygous k-mer pairs in Smudgeplot.

### Transposable elements in the genome of *Concholepas concholepas*

First, we mapped the set of clean and decominated PE reads to a newly assembled mitochondrial genome of *C. concholepas* (see below) with the program HISAT2 v2.2.1 ([52] Kim et al. 2019) and used only those reads that did not map to the mitochondrial genome (*n* = 61,865,612 PE reads) for the discovery, annotation, and quantification of repetitive elements in the nuclear genome of *C. concholepas* using the program dnaPipeTE v1.4c ( [53] Goubert et al. 2015, [54] Goubert 2022). Using low-coverage Illumina datasets, DnaPipeTE assembles repetitive elements and then annotates them based on homology with the program RepeatMasker (www.repeatmasker.org). Finally, DnaPipeTE maps a random sample of the reads onto the assembled repetitive elements to quantify their abundance. We executed DnaPipeTE with two iterations of the assembler Trinity using independent read sets, sampled at 0.25X, each time ([54] Goubert et al. 2022) and the Protostomia-specific database of transposable elements from the Dfam consortium (https://www.dfam.org/—[55] Hubley et al., 2016). Lastly, we retrieved the transposable elements landscape of *C. concholepas* which dnaPipeTE estimated by calculating and plotting the (blastn) divergence between transposable elements copies in the genomes (estimated from reads) and their respective assembled consensus sequences ([55] Goubert 2022).

### Nuclear ribosomal cassette in *Concholepas concholepas*

We assembled the 45S rRNA DNA of *C. concholepas* using the program TAREAN (tandem repeat analyzer—[56] Novak et al. 2017) as implemented in the pipeline RepeatRepeatExplorer v.2.3.8 ([56, 57] Novak et al. 2013, Novak et al. 2020) available in the platform Galaxy (http://repeatexplorer.org/). TAREAN identifies and assembles satellite DNA and nuclear ribosomal genes directly from unassembled short reads employing graph-based sequence clustering. Consensus sequences of repeat monomers are then reconstructed from the most frequent k-mers obtained by decomposing read sequences from corresponding clusters ([57] Novak et al. 2017). In TAREAN, all parameters were set to default values during the run. Following Tucker et al. (2023) [58], the exact coding positions of the 18S, 5.8S, and 28S nuclear rDNAs and the boundaries of the ITS1, ITS2, 5′ ETS, and 3′ ETS in the assembled operon were determined with the programs RNAmmer 1.2 ([59] Lagesen et al. 2007) using the eukaryote database, Infernal 1.0.2 ([60] Nawrocki et al. 2009) using a subset of Rfam 10.0 5.8S rRNA models, and ITSx v. 1.1b1 ([61] Bengtsson-Palme et al. 2013).

### Mitochondrial genome assembly and characterization in *Concholepas concholepas*

We used the program GetOrganelle v1.6.4 ([62] Jin et al., 2020) to de novo assemble the mitochondrial genome of *C. concholepas* using the totality of the raw Illumina reads. The mitochondrial genome of the cofamilial *Rapana venosa* (with GenBank accession number MZ435265) was used as a ‘seed’ during the assembly run that utilized k-mer sizes of 21, 55, 85, and 115 ([62] Jin et al., 2020).

The pipeline MITOS2 (http://mitos2.bioinf.uni-leipzig.de—[63] Donath et al. 2019) was used for the in silico annotation of the newly assembled mitochondrial genome and manual curation (i.e., readjustments to the start and stop codons of the protein coding genes [PCGs]) was conducted using the software MEGA X ([64] Kumar et al. 2018) and the web server translation tool ExPASy (https://web.expasy.org/translate/—[65] Gasteiger et al. 2003).

The web server Chloroplot (https://irscope.shinyapps.io/Chloroplot/—[66] Zheng et al. 2020) was used to render the studied mitochondrial genome as a circular map. Nucleotide usage for the entire mitochondrial genome was estimated using the software MEGA X. The codon usage of all PCGs was calculated using the codon usage tool in the web server Sequence Manipulation Suite (https://www.bioinformatics.org/sms2/codon_usage.html—[67] Stothard et al. 2000). Relative synonymous codon usage (RSCU) was calculated with the tool EZcodon as implemented in the web server EZmito (http://ezmito.unisi.it/—[68] Cucini et al. 2021).

We conducted an analysis of selective pressures for each mitochondrial PCG. For this purpose, the software KaKs_calculator 2.0 ([69] Wang et al. 2010) was used to calculate the number of nonsynonymous substitutions per nonsynonymous site dN, the number of synonymous substitutions per synonymous site dS, and the ratio ω = dN/dS for each PCG. During calculations, we used the γ-MYN model to account for mutation rate variance along the studied sequences and the cofamilial *Rapana venosa* as an outgroup (GenBank accession number KM213962). The observed ω ratio is expected to be equal to 1, < 1, or > 1, if a particular PCG is exposed to neutral selection, purifying (negative), or diversifying (positive) selection, respectively.

A relatively short non-coding region of the studied mitochondrial genome, its putative control region (CR), was described in detail. First, the tool Tandem Repeats Finder (https://tandem.bu.edu/trf/trf.html—[70] Benson et al. 1999) was used to determine the presence of tandem repeats in this region. Second, Simple Sequence Repeats (SSRs or microsatellites) were detected in this region using the web server Microsatellites Repeats Finder (http://insilico.ehu.es/mini_tools/microsatellites/—[71] Bikandi et al. 2004). Lastly, the web server RNAfold (http://rna.tbi.univie.ac.at//cgi-bin/RNAWebSuite/RNAfold.cgi—[72] Gruber et al. 2008) was used to probe for the presence of ‘hairpins’ or ‘stem and loops’ along the studied non-coding sequence.

### Phylogenetic position of *Concholepas concholepas* in the family Muricidae

We tested the phylogenetic position of *C. concholepas* in the family Muricidae based on the phylogenetic signal from translated PCGs. A maximum likelihood (ML) phylogenetic analysis was conducted using the newly assembled mitochondrial genome of *C. concholepas*, a second mitochondrial genome of *C. concholepas* already available in GenBank (JQ446041), and those of 32 cofamilial species with mitochondrial genomes available in GenBank. The analysis used 11 other species belonging to other neogastropod families as outgroups. First, each set of PCG nucleotide sequences was translated to amino acids and then aligned with the program Clustal Omega ([73] Sievers and Higgins, 2014). Next, we eliminated poorly aligned regions with the program trimAl ([74] Capella-Gutiérrez et al., 2009) in each PCG alignment and used the program ProtTest ([75] Darriba et al., 2011) to partition the dataset and select the best fitting models of sequence evolution for each partition. Lastly, a ML analysis was conducted in the web server IQ-TREE version 1.6.10 ([76] Nguyen et al., 2015) with the concatenated but partitioned PCG amino acid alignment. The robustness of the ML tree topology was assessed by 1,000 bootstrap iterations of the observed data as in [58] Tucker et al. (2023).

### Supplementary Information

**Additional file 1: Table S1.** Codon usage in mitochondrial protein coding genes of *Concholepas concholepas* (n residuals = 11,085). **Figure S1.** Stem and loop structure find in the relatively short non-coding putative Control Region of *Concholepas concholepas*.

## Data Availability

The whole-genome sequencing data are available in the NCBI Sequence Read Archive (SRA) repository (Bioproject: PRJNA996197; BioSample: SAMN36527401; SRA accession number: SRR25338493; Assembly: JAUZNI000000000) at NCBI’s GenBank.

## Abbreviations

*Ala*: Alanine amino acid
*Arg*: Arginine amino acid
*bv*: Bootstrap value
*cm*: Centimeters
*cob*: Cytochrome b subunit of the cytochrome bc1 complex
*cox*: Subunits of the cytochrome c oxidase
*CR*: Control Region
*d*_*N*_: Non-synonymous substituions
*d*_*S*_: Synonymous substitutions
*Gln*: Glutamine aminoacid
*H*: Heavy strand
Ka: Non-synonymous substitutions per non-synonymous site
Ks: Synonymous substitutions per synonymous site
*Leu*: Leucine aminoacid
*Lys*: Lysine amino acid
*m*: Meters
*mtDNA*: Mitochondrial genome
*nad*: Subunits of the NADH dehydrogenase complex
*NCBI*: National Center for Biotechnology Information
*PCG’s*: Protein coding genes
*PE*: Paired-end
*rRNA*: Ribosomal genes
*rrnL*: 16S ribosomal RNA
*rrnS*: 12S ribosomal RNA
*RSCU*: Relative Synonymous Codon Usage
*Ser*: Serine amino acid
*Thr*: Threonine amino acid
*tRNA*: Transfer RNA genes
*Trp*: Tryptophan amino acid
*Val*: Valine aminoacid
*ω* = *d*_*N*_*/d*_*S*_ = *Ka/Ks*: The ratio of non-synonymous to synonymous substitutions
