# Supplementary material for: Insights into the genome of the ‘Loco’ Concholepas concholepas (Gastropoda: Muricidae) from low-coverage short-read sequencing: genome size, ploidy, transposable elements, nuclear RNA gene operon, mitochondrial genome, and phylogenetic placement in the family Muricidae
Source: BMC Genomics. 2024 Jan 19;25:77. doi: 10.1186/s12864-023-09953-7 (PMC10797722; doi:10.1186/s12864-023-09953-7)
Supplement: Supplementary file 1 — Additional file 1: Table S1. Codon usage in mitochondrial protein coding genes of Concholepas concholepas (n residuals = 11,085). Figure S1. Stem and loop structure find in the relatively short non-coding putative Control Region of Concholepas concholepas. [file 12864_2023_9953_MOESM1_ESM.docx]

**Supplementary Materials**

**Insights into the genome of the ‘Loco’ *Concholepas concholepas* (Gastropoda: Muricidae) from low-coverage short-read sequencing: Genome size, ploidy, transposable elements, nuclear RNA gene operon, mitochondrial genome, and phylogenetic placement in the family Muricidae.**

J. Antonio Baeza ^1,2.3 *^ M. Teresa González ^4^, Julia D. Sigwart^5^, Carola Greve^6^, Stacy Pirro^7^

^1^ Department of Biological Sciences, Clemson University, Clemson, SC, USA

^2^ Departamento de Biología Marina, Universidad Catolica del Norte, Coquimbo, Chile

^3^ Smithsonian Marine Station at Fort Pierce, Smithsonian Institution, Fort Pierce, FL, USA

^4^ Instituto de Ciencias Naturales Alexander von Humboldt, Facultad de Ciencias del Mar y Recursos Biológicos, Universidad de Antofagasta, Angamos 601, Antofagasta, Chile.

^5^ Senckenberg Research Institute and Museum,Marine Zoology Department, Frankfurt, Germany

^6^LOEWE -Centre for Translational Biodiversity Genomics (LOEWE-TBG), Senckenberganlage 25, Frankfurt am Main, Germany

^7^ Iridian Genomes, Bethesda, Maryland, USA

* Corresponding author.

E-mail address: baeza.antonio@gmail.com (J.A. Baeza).

**Table S1**. Codon usage in mitochondrial protein coding genes of *Concholepas concholepas* (n residuals = 11,085)

_____________________________________

AmAcid Codon Number /1000 Fraction

______________________________________

Ala GCG 16.00 4.33 0.06

Ala GCA 64.00 17.32 0.26

Ala GCT 120.00 32.48 0.48

Ala GCC 49.00 13.26 0.20

Cys TGT 33.00 8.93 0.79

Cys TGC 9.00 2.44 0.21

Asp GAT 39.00 10.55 0.55

Asp GAC 32.00 8.66 0.45

Glu GAG 44.00 11.91 0.48

Glu GAA 47.00 12.72 0.52

Phe TTT 236.00 63.87 0.74

Phe TTC 85.00 23.00 0.26

Gly GGG 63.00 17.05 0.25

Gly GGA 103.00 27.88 0.41

Gly GGT 54.00 14.61 0.22

Gly GGC 31.00 8.39 0.12

His CAT 34.00 9.20 0.42

His CAC 47.00 12.72 0.58

Ile ATT 220.00 59.54 0.79

Ile ATC 58.00 15.70 0.21

Lys AAG 32.00 8.66 0.37

Lys AAA 54.00 14.61 0.63

Leu TTG 85.00 23.00 0.15

Leu TTA 203.00 54.94 0.35

Leu CTG 36.00 9.74 0.06

Leu CTA 101.00 27.33 0.17

Leu CTT 122.00 33.02 0.21

Leu CTC 37.00 10.01 0.06

Met ATG 78.00 21.11 0.42

Met ATA 108.00 29.23 0.58

Asn AAT 83.00 22.46 0.70

Asn AAC 36.00 9.74 0.30

Pro CCG 18.00 4.87 0.12

Pro CCA 28.00 7.58 0.19

Pro CCT 72.00 19.49 0.49

Pro CCC 28.00 7.58 0.19

Gln CAG 36.00 9.74 0.49

Gln CAA 37.00 10.01 0.51

Arg CGG 14.00 3.79 0.24

Arg CGA 30.00 8.12 0.51

Arg CGT 10.00 2.71 0.17

Arg CGC 5.00 1.35 0.08

Ser AGG 27.00 7.31 0.07

Ser AGA 58.00 15.70 0.16

Ser AGT 35.00 9.47 0.09

Ser AGC 27.00 7.31 0.07

Ser TCG 13.00 3.52 0.04

Ser TCA 46.00 12.45 0.12

Ser TCT 135.00 36.54 0.36

Ser TCC 30.00 8.12 0.08

Thr ACG 16.00 4.33 0.10

Thr ACA 44.00 11.91 0.27

Thr ACT 76.00 20.57 0.46

Thr ACC 28.00 7.58 0.17

Val GTG 30.00 8.12 0.11

Val GTA 88.00 23.82 0.33

Val GTT 120.00 32.48 0.45

Val GTC 31.00 8.39 0.12

Trp TGG 28.00 7.58 0.26

Trp TGA 78.00 21.11 0.74

Tyr TAT 84.00 22.73 0.62

Tyr TAC 51.00 13.80 0.38

End TAG 3.00 0.81 0.23

End TAA 10.00 2.71 0.77

_____________________________________


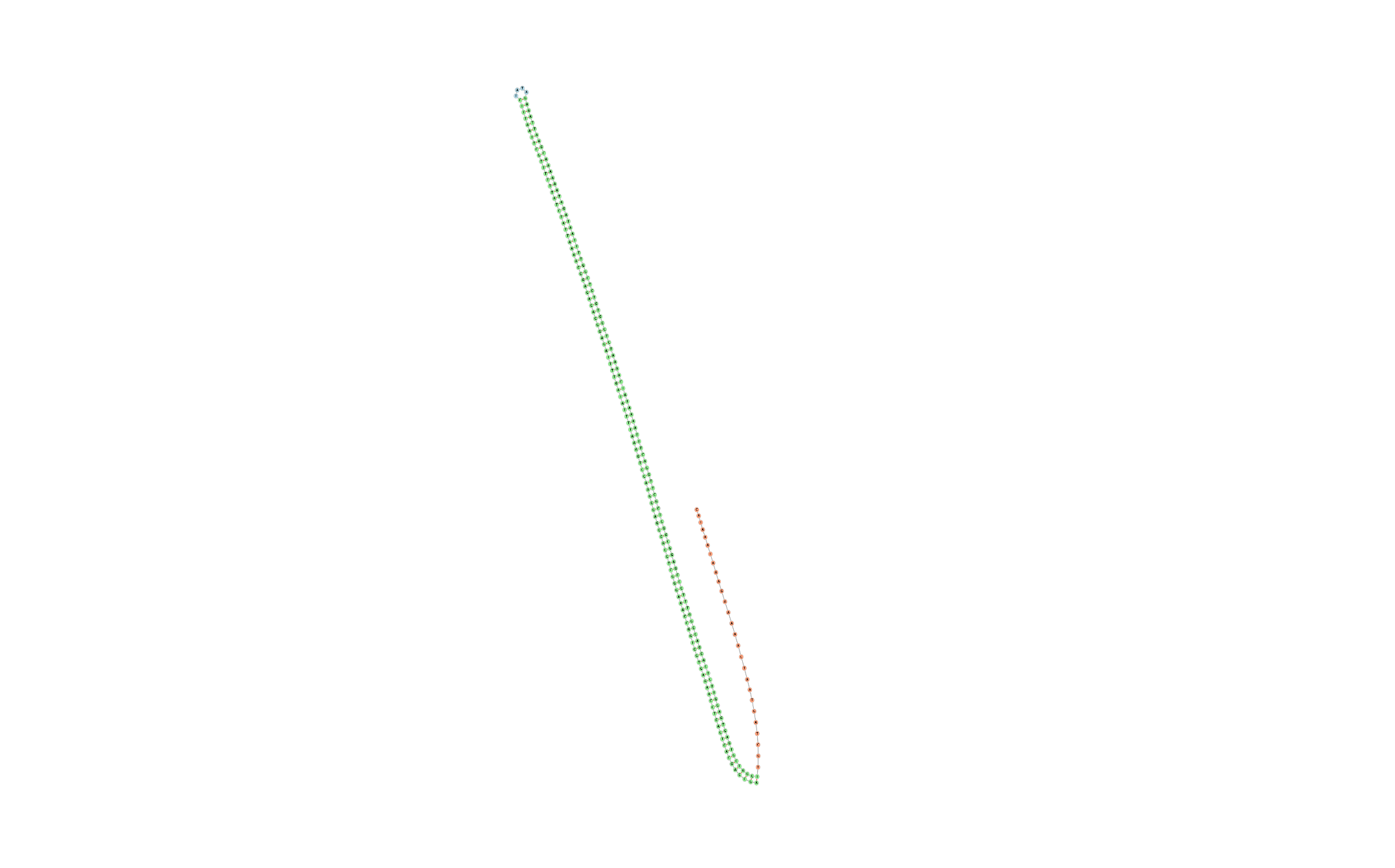


**Figure S1.** Stem and loop structure find in the relatively short non-coding putative Control Region of *Concholepas concholepas*.
